# Synchronized drowsiness monitoring and simulated driving performance data under 50-hr sleep deprivation: A double-blind placebo-controlled caffeine intervention

**DOI:** 10.1016/j.dib.2018.06.006

**Published:** 2018-06-09

**Authors:** E. Aidman, K. Johnson, B.L. Hoggan, J. Fidock, G.M. Paech, C.B. Della Vedova, M. Pajcin, C. Grant, G. Kamimori, E. Mitchelson, S. Banks

**Affiliations:** a Defence Science and Technology Group, Land Division, Edinburgh, Australia; b University of South Australia, School of Psychology, Social Work and Social Policy, Centre for Sleep Research, Adelaide, Australia; c University of South Australia, School of Pharmacy and Medical Sciences, Adelaide, Australia; d Behavioral Biology Branch, Center for Military Psychiatry and Neuroscience Research, Walter Reed Army Institute of Research, Silver Spring, United States

## Abstract

This paper presents the 60-s time-resolution segment from our 50-h total sleep deprivation (TSD) dataset (Aidman et al., 2018) [1] that captures minute-by-minute dynamics of driving performance (lane keeping and speed variability) along with objective, oculography-derived drowsiness estimates synchronised to the same 1-min driving epochs. Eleven participants (5 females, aged 18–28) were randomised into caffeine (administered in four 200 mg doses via chewing gum in the early morning hours) or placebo groups. Every three hours they performed a 40 min simulated drive in a medium fidelity driving simulator, while their drowsiness was continuously measured with a spectacle frame-mounted infra-red alertness monitoring system. The dataset covers 15 driving periods of 40 min each, and thus contains over 600 data points of paired data per participant. The 1-min time resolution enables detailed time-series analyses of both time-since-wake and time-on-task performance dynamics and associated drowsiness levels. It also enables direct examination of the relationships between drowsiness and task performance measures. The question of how these relationships might change under various intervention conditions (caffeine in our case) seems worth further investigation.

**Specifications Table**

**Table:** 

Subject area	Psychology & Human factors
More specific subject area	Operator fatigue countermeasures
Type of data	Simulator-derived driving performance data synchronised with drowsiness biomarker data
How data was acquired	1. Simulated driving protocol rendered in Virtual BattleSpace-2 software (VBS-2; Bohemia, USA) was used to assess driving performance with measures of lane positioning variability and speed variability 2. Drowsiness was measured with spectacle frame-mounted infra-red oculography - Optalert Alertness Monitoring System (OAMS) producing a Johns Drowsiness Scale (JDS) score every 60 s [2]
Data format	1. JDS scores generated every 60 s 2. VBS-generated lateral lane positioning variability (SDlat) and speed variability (SDS) synchronised to JDS scores in 60-s epochs. 3. Long format with approximately 600 paired data points per participant
Experimental factors	1. 50-h total sleep deprivation 2. Randomized double-blind placebo-controlled caffeine intervention 3. Driving tasks repeated every 3 h: 15 repeats in total under accumulated sleep loss 4. Participants were screened for pre-existing medical conditions (including sleep disturbances), tobacco and recreational drug use, recent time-zone travel and shift-work
Experimental features	1. 50-h total sleep deprivation protocol 2. Randomized double-blind placebo-controlled caffeine intervention: 4 oral doses of either caffeinated gum pellets (200 mg/dose) or non-caffeinated placebo gum every two hours (01:00, 03:00, 05:00, 07:00) during both nights of sleep deprivation 3. Driving tasks: 40-min monotonous driving tasks in a medium-fidelity moving-base driving simulator, separated by 140 min of un-related activities (three-hour cycles) 4. Drowsiness and driving performance continuously measured in 60-s epochs
Data source location	Adelaide, South Australia
Data accessibility	The data is with this article

**Value of the data**

• Fatigue countermeasures are typically evaluated with performance metrics aggregated over sustained work periods [3], [4]. Our data enables the assessment of countermeasure effects on performance dynamics within the task by combining a double-blind placebo-controlled intervention with synchronised drowsiness and performance monitoring.
• This data structure enables detailed analyses of performance dynamics and associated drowsiness levels. Both global decline slopes (time-since-wake) and time-on-task effects within individual drives can be analysed, as well as their interaction. These analyses go beyond those reported by Aidman et al. [1].
• Our data structure also enables direct examination of the relationship between drowsiness and task performance in order to investigate the interaction between distal (time-since-wake) and proximal (drowsiness) causes of performance fatigue. These interactions can be analysed for various performance outcomes – from driving (this dataset) to standardised cognitive testing.
• Our data illustrates a method of validating ocular measures of drowsiness by analysing its real-time associations with task performance, under sleep deprivation or any other intervention of choice
• More broadly, our dataset, and the experimental design it reflects, combining synchronised biomarker and performance measurement with randomised treatment group allocation, offers a more time-sensitive method of evaluating interventions in human factors research – moving beyond gross performance evaluations to assessing performance dynamics and detecting time-critical risk factors.

## 1 Data

This paper presents the 60-s time-resolution segment from our 50-h total sleep deprivation (TSD) dataset [1], [4] that captures minute-by-minute dynamics of driving performance (lane keeping and speed variability) along with objective, oculography-derived drowsiness estimates synchronised to the same 1-min driving epochs. The dataset contains 6170 rows in a long format, with 40+ paired data points per drive (“Time on task” column, in 1-min intervals).

## 2 Experimental design, materials and methods

### 2.1 Participants

As part of a larger study [5], 24 young healthy adults, aged 18 to 28 years (*M*=22.46±2.73), completed fifteen identical 40-min simulated driving tasks across the 50-h TSD protocol (see Fig. 1 in [1]). They were screened for abnormal sleep-wake patterns (validated through sleep diaries and actigraphy), high caffeine consumption (>250 mg/day), excessive body weight (BMI > 30), smoking, recreational drug use and ill-health (assessed by a general health questionnaire and blood toxicology screen) and for shift-work or trans-meridian travel in the three months prior to the study. The data represent a sub-sample of 11 from this larger study (6 male) who completed the drowsiness monitoring protocol throughout their driving tasks (see Fig. 2 in [1] for other sampling details). The University of South Australia Human Research Ethics Committee approved this study prior to recruiting participants, who gave their written consent prior to commencing their participation.

### 2.2 Procedure

Participants were required to maintain a minimum of 8 h time in bed (TIB) for 7 days prior to the study (verified via sleep diaries and actigraphy) and to abstain from caffeine and alcohol, as one week of caffeine abstinence is known to sharpen the effects of its re-introduction,while having minimal withdrawal effects [6]. The experiment took place in a controlled sleep laboratory environment, with ambient temperature maintained at 22±1 °C, and light levels at ≤50 lx during wakefulness and ≤1 lx during sleep. A full day prior to the commencement of the TSD protocol was allocated for habituation, task training and equipment calibration. The protocol commenced at 0800, following a 10-h sleep opportunity (2200-0800), and included a 40-min simulated driving task performed every three hours in a medium-fidelity driving simulator (15 drives in total over the 50-h protocol). Participants׳ drowsiness was continuously measured by spectacle frame-mounted infra-red oculography – the Optalert Alertness Monitoring System (OAMS, [2]).

In a randomised double-blind, placebo-controlled design, the treatment group (*n*=5) received four doses of 200 mg caffeine via gum pellets (Military Energy Gum; MarketRight, Plano, IL, USA) every two hours (0100, 0300, 0500, 0700) during each of the two nights of TSD. The placebo group (*n*=6) consumed non-caffeinated chewing gum that was identical in appearance, texture and flavour, at the same times when the treatment group consumed caffeine. All participants were instructed to chew the pellets for a minimum of 5 min, as this is known to be sufficient for releasing 85% of the dose [7].

### 2.3 Simulated driving task

A medium-fidelity motion-base driving simulator comprised a 42-inch screen positioned 1.2 m in front of the steering wheel, driving controls and driver׳s seat mounted on a motion frame equipped with D-BOX linear actuators producing three types of motion: up to 4° of left/right rotation (roll), up to 4° of forward-backward rotation (pitch) and up to 40 mm of vertical movement (heave). The resulting motion averaged 0.08 m/s^2^ (calculated as the root mean square addition of the three axes and classed to be within safe limits under the “Whole Body Vibration” standard AS2670.1).

The audio-visual streams and motion actuators were controlled by Virtual BattleSpace-2 (VBS-2; Bohemia, USA). The driving scenario, developed under VBS-2 simulation environment, required driving continuously along a slightly curved section of two-lane rural highway at dusk, with no features or other vehicles in the environment. Participants were instructed to stay in the left-hand lane and travel at a constant speed of 80 km/h. When the tires of the simulated vehicle left the road, its speed decreased and the motion actuators would mimic an unsealed road. When the vehicle׳s lane deviation exceeded 2.5 m (the full width of the vehicle), the simulation registered a crash and reset the car to a stationary central position requiring a manual restart.

## 3 Measures

### 3.1 Drowsiness: Johns Drowsiness Scale

The OAMS system uses an infra-red emitter and sensor mounted on a spectacle frame to continuously monitor eye and eyelid movements during blinks – including their timing, duration, and velocity. These ocular parameters are then combined to quantify drowsiness levels in the form of the Johns Drowsiness Scale (JDS) scores [8]. The OAMS estimates drowsiness by generating a JDS score over regular time epochs. Epoch duration in this study was 60 s, thus enabling sufficient time resolution with 40 data points fitting each 40-min drive period. The JDS scores range from 0 (very alert) to 10 (very drowsy) with a JDS score between 0 and 4.4 (inclusive) indicating a relatively low risk level of drowsiness. A score of 4.5 to 4.9 (inclusive) reflects a moderate drowsiness risk and a score above 5.0 indicates a critical level of drowsiness risk.

The JDS has been shown to have high test-retest reliability across different levels of drowsiness (*r*=.80; [9]) and to reliably co-vary with circadian and homeostatic sleep pressure [10], [11], [12], [13], [14].

Participants wore the Optalert glasses for approximately 5 min prior to each drive to allow the OAMS to begin registering JDS scores at the start of the drive. Each JDS score was time-stamped to enable synchronisation with simulated driving performance data.

### 3.2 Driving performance

Driving performance was measured using standard deviation of lateral lane position (SDlat) in metres (m) and standard deviation of speed (SDS) in kilometres per hour (km/h). These measures were aggregated over one-minute intervals to coincide with the same 60-s epochs from which our OAMS generated the Johns Drowsiness Scores.

#### 3.2.1 Lane keeping

Standard deviation of lateral lane position (SDlat) is a well-established, sensitive and objective driving performance indicator in sleepiness studies [15], [16], [17], [18], [19], [20], [21]. Lateral position in the current dataset was defined as the distance from the centre of the road.

#### 3.2.2 Speed maintenance

Driving speed variability is known to be sensitive to sleep loss [20], [22], [23]. We used standard deviation of speed as a measure of speed variability.

Both driving performance variables (SDlat and SDS) were positively skewed. They were natural log-transformed for normality. These log-transformed values have been added to the dataset.
