# Supplementary material for: Synchronized drowsiness monitoring and simulated driving performance data under 50-hr sleep deprivation: A double-blind placebo-controlled caffeine intervention
Source: Data Brief. 2018 Jun 9;19:1335–40. doi: 10.1016/j.dib.2018.06.006 (PMC6141128; doi:10.1016/j.dib.2018.06.006)
Supplement: Supplementary file 1 — Supplementary material [file mmc1.docx]

Declaration of interests is identical to the Acknowledgements section in the paper (copied below).

**Acknowledgements**

The authors are grateful to Philip Jacques, Cassie Hilditch, Stephanie Centofanti and Stephanie Eonta for assistance with equipment setup and data collection, and to Gary Wittert for being the physician of record on this study. The research was funded by the Australian Defence Project Land 121 Phase 4 under the Research Agreement 558023. This work was also supported by the US Army Medical Research and Material Command. The opinions contained herein are the private views of the authors, and are not to be construed as official, or as reflecting true views of the United States Department of the Army or the United States Department of Defense.
